# The Role of Output Monitoring and Review on Teacher Job Performance in Secondary Schools: Evidence from Kasese District, Uganda

**DOI:** 10.12688/f1000research.166552.1

**Published:** 2025-08-04

**Authors:** Asiati Mbabazi, Zulaiha Lawal Bagiwa, Tukur Muhammad, Lucy Aja

**Affiliations:** 1 Département of Foundations, Faculty of Education, Kampala International University - Western Campus, Bushenyi, Western Region, Uganda; 2 Department of Science Education, Faculty of Education, Kampala International University - Western Campus, Bushenyi, Western Region, Uganda

**Keywords:** Teacher performance, Output monitoring, Output review, Performance evaluation, Secondary schools, Uganda

## Abstract

**Background:**

Teacher job performance is an important factor influencing the quality of education and student learning outcomes. Effective output monitoring and review ensure teachers adhere to instructional standards. This study examines the impact of output monitoring and review on teacher job performance in secondary schools in Kasese District.

**Methods:**

A cross-sectional research design was used, integrating quantitative and qualitative approaches. The study targeted 675 participants, including head teachers, deputy head teachers, directors of studies, and teachers, with a final sample of 245 respondents selected through stratified random and purposive sampling. Data were collected using structured questionnaires and interview guides and analyzed using SPSS version 26, employing descriptive statistics, Pearson correlation, multiple regression, and Bayesian inference to test the study hypotheses.

**Findings:**

The results rejected the null hypotheses, confirming that both output monitoring (R ^2^ = 0.589, F = 14.071, p < 0.05) and output review (R ^2^ = 0.715, F = 16.648, p < 0.05) significantly influence teacher job performance. Teachers who received structured feedback and periodic performance evaluations demonstrated higher engagement, improved instructional quality, and increased motivation. The Bayesian factor inference confirmed the robustness of these findings. Pearson correlation analysis revealed a moderate positive correlation between output monitoring (r = 0.521) and teacher job performance and a weak to moderate correlation for output review (r = 0.321).

**Conclusion:**

Schools should strengthen performance monitoring frameworks, establish objective and transparent evaluation criteria, and provide regular, structured feedback linked to professional development initiatives. Integrating digital performance tracking tools can enhance efficiency and transparency in teacher evaluation, ultimately improving educational outcomes.

## Key findings of the study

○ Output monitoring significantly enhances teacher accountability, instructional quality, and engagement, as indicated by a moderate positive contribution to teacher job performance (R ^2^ = 0.589, F = 14.071, p < 0.05).
○ Output review exerts a stronger influence on teacher job performance compared to output monitoring, accounting for a higher variance in teacher effectiveness (R ^2^ = 0.715, F = 16.648, p < 0.05).
○ Teachers who receive structured performance evaluations and regular feedback demonstrate higher levels of motivation, improved classroom management, and enhanced instructional effectiveness.
○ The hypothesis (H _0_1) stating that output monitoring has no significant influence on teacher job performance was statistically rejected, confirming that structured monitoring contributes meaningfully to improving teacher effectiveness.
○ The hypothesis (H _0_2) stating that output review has no significant influence on teacher job performance was also rejected, emphasizing the important role of structured performance evaluation in driving teacher productivity.
○ Bayesian inference analysis further validated that both output monitoring and review mechanisms serve as key predictors of teacher job output, reinforcing their importance in school management practices.
○ Schools that implement well-structured and transparent evaluation systems report significantly higher levels of teacher performance, leading to improved student learning outcomes.

## Introduction

Teacher job performance plays a crucial role in the overall effectiveness of the education system, directly influencing student learning outcomes and institutional success. ^1^ Schools rely on structured performance management systems to ensure that teachers meet the required educational standards and continuously improve their instructional practices. Two critical components of teacher performance management are output monitoring and output review. ^2^ Output monitoring refers to the continuous assessment of teachers’ instructional activities, classroom engagement, and adherence to established teaching standards. While output review involves the systematic evaluation of teachers’ performance through structured feedback, appraisal reports, and performance discussions. ^3^

Globally, performance monitoring and review have been widely recognized as essential strategies for enhancing teacher effectiveness. ^4^ Countries with well-developed education systems, such as Finland, Singapore, and Canada, have established comprehensive teacher evaluation frameworks that emphasize ongoing monitoring and constructive feedback to drive professional growth. ^5^ Research has shown that effective teacher monitoring enhances accountability, improves instructional quality, and fosters professional development. Similarly, output reviews have been associated with increased teacher motivation, better classroom management, and improved student performance. ^6^

In Sub-Saharan Africa, many education systems face challenges in implementing robust performance appraisal mechanisms. The main reasons include limited resources, administrative inefficiencies, and lack of structured teacher evaluation policies. ^7^ Studies indicate that many schools in the region lack effective performance-tracking systems, leading to inconsistencies in teacher supervision and evaluation. As a result, many teachers operate with minimal guidance, affecting their motivation and overall job effectiveness. ^8^

Uganda’s education system has undergone significant expansion and reform over the past decades, including the introduction of Universal Secondary Education (USE) in 2007 to increase access to quality education. ^9^ Despite these efforts, challenges such as high teacher attrition, inconsistent monitoring practices, and inadequate performance appraisal mechanisms persist, affecting the quality of teaching in secondary schools. ^10, 11^ This has led many schools to rely on ad-hoc supervision methods, resulting in subjective assessments that lack meaningful feedback for professional growth. Additionally, teacher absenteeism, inadequate lesson preparation, and low motivation levels remain prevalent due to weak performance appraisal mechanisms. Existing research on teacher evaluation in Uganda has primarily focused on student academic performance and school leadership, with limited emphasis on the role of output monitoring and review in shaping teacher job performance. ^12^ Thus, there is a knowledge gap on how structured monitoring and review systems impact teacher effectiveness, classroom engagement, and instructional quality in secondary schools in Kasese District.

Kasese District, located in Western Uganda, exemplifies these challenges, as schools struggle with large class sizes, disparities in performance evaluations, and difficulties in maintaining teacher accountability. ^13^ These challenges highlight the need for an effective system of output monitoring and review to enhance teacher job performance and improve educational outcomes. This study aims to investigate the impact of output monitoring and review on teacher job performance in secondary schools within Kasese District.

## Methodology

### Study design

This study employed a cross-sectional research design to investigate the impact of output monitoring and output review on teachers’ job performance in secondary schools in Kasese District. A cross-sectional design was selected because it allows for data collection at a single point, providing a snapshot of the current state of teacher job output regarding monitoring and review mechanisms.

### Research approach

The study adopted a quantitative research approach. Integrating quantitative methodologies to help comprehensively understand the research problem.

### Study area

The study was conducted in Kasese District, Uganda, an area characterized by diverse educational settings, including government-aided and private secondary schools situated in urban, semi-urban, and rural locations. The district’s educational landscape is marked by high teacher attrition, large class sizes, and disparities in performance evaluation practices, making it a suitable context for investigating the impact of output monitoring and review mechanisms.

### Target population

The study targeted a population of 675 participants, comprising head teachers, deputy head teachers, directors of studies, and teachers in Kasese District. This population was selected based on their direct involvement in teaching and school administration, making them well-positioned to provide relevant information regarding the impact of output monitoring and review on teacher job performance. The distribution of the study population included 23 head teachers, 23 deputy head teachers, 23 directors of studies, and 606 teachers.

### Sample size determination

The sample size was determined using Cochran’s (1977) formula, which is appropriate for large populations and ensures that the sample is representative. The final sample comprised 245 respondents, including all head teachers, deputy head teachers, and directors of studies, while teachers were selected using stratified random sampling to ensure adequate representation from different school types and locations.

### Data collection tool

Data collection involved the use of a structured questionnaire. The questionnaire, designed for teachers, deputy head teachers, and directors of studies, included closed-ended questions measured on a 5-point Likert scale to capture respondents’ perceptions of output monitoring, review processes, and their impact on job performance. The structured questionnaire ensured uniformity in responses.

### Validation of instrument

The research instruments underwent validity and reliability testing to ensure quality control, and several measures were taken, including content validity was established through expert review, where three education specialists evaluated the questionnaire’s relevance and clarity, with a content validity index (CVI) of ≥0.7 considered acceptable.

### Reliability of instrument

A pilot study was conducted in three secondary schools that were not part of the main study to refine ambiguous questions and ensure clarity. Reliability testing was performed using Cronbach’s Alpha (≥0.7 threshold) to measure internal consistency, ensuring that the questionnaire would produce consistent results when administered to similar populations.

### Hypothesis statement

This study examined the influence of structured output planning, monitoring, and review on teacher job performance in secondary schools in Kasese District. To statistically validate these relationships, the following hypotheses were tested at a 0.05 level of significance using Pearson correlation and multiple regression analysis:

○ **H _01_:** Output monitoring has no significant effect on teachers’ job performance.
○ **H _02_:** Output review does not significantly impact teachers’ job performance.

The hypotheses were assessed using SPSS version 26, with descriptive statistics used to summarize responses, correlation analysis to measure associations between variables, and regression analysis to determine the predictive strength of output monitoring and review on teacher job performance. Bayesian inference was further applied to reinforce the reliability of the statistical findings. Hypotheses were rejected or retained based on the significance values (p < 0.05) and model fit statistics.

### Statistical analysis

Data were analysed using SPSS version 26, applying both descriptive and inferential statistical methods to assess the relationship between structured output planning, monitoring, and review with teacher job performance. The descriptive analysis utilised measures of central tendency, including means and standard deviations, to analyse the responses on structured output planning, monitoring, and review. Pearson correlation was employed to determine the strength and direction of relationships between the key study variables. Multiple linear regression analysis was conducted to assess the predictive contribution of structured output planning, monitoring, and review to teacher job performance. Bayesian statistical techniques were used to provide additional validation for the regression models and confirm the robustness of the findings. All statistical tests were performed at a 0.05 level of significance, with assumptions of normality and homoscedasticity verified before conducting inferential analyses.

## Results

The results in Table 1 indicate a very high level of agreement among teachers regarding the effectiveness of output monitoring in secondary schools in Kasese District, with a pooled mean of 4.225. The majority of respondents strongly agreed or agreed that school management employs acceptable monitoring procedures (M = 4.48) and that supervision techniques are generally comfortable (M = 3.93). Teachers expressed comfort with their supervisors’ appraisal style (M = 4.22 **)**, and most acknowledged that monitoring has improved their teaching activities (M = 4.08). Lesson supervision was identified as a commonly used method for monitoring (M = 4.14). Commitment to work was perceived as a motivating factor (M = 4.18), and objectivity in monitoring activities was well-rated (M = 4.44). Additionally, teachers expressed high satisfaction with the monitoring methods used by their supervisors (M = 4.33). Results show that structured output monitoring has a moderately positive effect on teachers’ job output (R ^2^ = 0.589, F = 14.071, p < 0.05). A majority of teachers (pooled mean = 4.225) agreed that regular monitoring through lesson supervision, attendance tracking, and performance appraisals improved their instructional effectiveness.

**Table 1.** Descriptive statistical analysis for output monitoring in teachers' job output in secondary schools in Kasese district.

#	Statement	SA (5)	A (4)	N (3)	D (2)	SD (1)	$\text{Mean}\;\left(\bar{x}\right)$	Standard deviation(s)
OPM1	The management of my school utilizes acceptable monitoring procedures to carry out monitoring of me.	N=59 (24%)	N=169 (68.9%)	N=8 (3.3%)	N=1 (0.4%)	N=1 (0.4%)	4.48	0.585
OPM2	I am very much comfortable with the technique used in supervising me during …	N=93 (37.8%)	N=133 (54.4%)	N=17 (7.1%)	N=1 (0.4%)	N=1 (0.4%)	3.93	0.844
OPM3	I am comfortable with the style of my supervisor, as he appraises my job output.	N=101 (41.4%)	N=109 (44.4%)	N=36 (13.3%)	N=2 (3.7%)	N=1 (0.4%)	4.22	0.719
OPM4	The process of monitoring my output has helped me improve my teaching activities.	N=136 (55.6%)	N=73 (29.9%)	N=34 (13.9%)	N=1 (0.4%)	N=1 (0.4%)	4.08	0.737
OPM5	Most time, my supervisor employs the lesson supervision method to monitor my job output.	N=97 (39.4%)	N=111 (45.2%)	N=36 (14.1%)	N=2 (0.8%)	N=1 (0.4%)	4.14	0.804
OPM6	Using a commitment to work as a yardstick of output monitoring encourages me to do more.	N=97 (39.8%)	N=118 (48.1%)	N=27 (11.2%)	N=1 (0.4%)	N=1 (0.4%)	4.18	0.778
OPM7	All monitoring activities are done based on the principle of objectivity regarding the set targets and goals of the school.	N=125 (51.1%)	N=100 (40.7%)	N=18 (7.5%)	N=2 (0.8%)	N=1 (0.4%)	4.44	0.598
OPM8	I am very pleased with the method of monitoring my supervisor …. materials.	N=142 (58.1%)	N=100 (40.7%)	N=1 (0.4%)	N=1 (0.4%)	N=1 (0.4%)	4.33	0.561
	Pooled mean	N=106 (43.3%)	N=114 (46.6%)	N=22 (8.8%)	N=3 (1.3%)	N=1 (0.4%)	**4.225**	

Key: (OPM)= output monitoring, 2.00-2.99 Low, 3.00-3.99 high, and 4.00-4.99 Very high Key: SA (5) =Strongly Agree, A (4) =Agree, N (3) =Neutral in Agreement, D (2) =Disagree, SD (1) =Strongly Disagree.

The results of Table 2 show a moderate positive correlation (r = 0.521) between teachers’ job output and output planning. Pearson correlation analysis (r = 0.521) confirmed a moderate positive association between output monitoring and teachers’ job performance, highlighting that objective and structured monitoring enhances accountability and motivation.

**Table 2.** Bayes’ factor inference on pairwise correlations.

		Teachers job output	Output planning
Teachers Job Output	Pearson Correlation	1	0.521
	^a^Bayes Factor		0.00
	N	245	245
Output Planning	Pearson Correlation	0.521	1
	^a^Bayes Factor	0.00	
	N	245	245

^a^ Bayes factor: Inference on Pairwise Correlations.

The regression analysis for this study in Table 3 shows that output monitoring variables moderately contribute 58.9% (R ^2^ = 0.589) towards the success of the teacher’s job output. Where $R^{2}=0.589$ , F=14.041 and the standard error of the estimate Se=0.1649 . The F-value (14.071) confirms the statistical significance of this relationship (F > 3.00)

**Table 3.** Summary of regression diagnostics.

Bayes factor	R	RSquare	AdjustedRSquare	Standard error of estimate	F
4.251E + 16	0.730	0.589	0.553	0.1649	14.071

The results in Table 4 show that the influence of output review on teachers’ job output in secondary schools in Kasese District was assessed, with results indicating a very high level of agreement (Mean = 4.23). A majority (92%) of teachers agreed or strongly agreed that output reviews provided constructive feedback, improvement opportunities, and alignment with performance standards. The highest-rated statements included constructive feedback from supervisors (Mean = 4.48) and agreement on action plans during reviews (Mean = 4.44).

**Table 4.** Descriptive statistical analysis on the influence of output review on teachers’ job output in secondary schools in Kasese district.

#	Statement	SA (5)	A (4)	N (3)	D (2)	SD (1)	$\text{Mean}\;\left(\bar{x}\right)$	Standard deviation(s)
OPR1	I have received several opportunities from my superiors after reviewing my performance output.	N=80 (32.8%)	N=145 (59.3%)	N=17 (7.1%)	N=1 (0.4%)	N=1 (0.4%)	4.48	0.585
OPR2	My superiors always review my output in line with the set performance standards of the school.	N=100 (40.7%)	N=127 (51.9%)	N=17 (7.1%)	N=0 (0.0%)	N=1 (0.4%)	3.93	0.844
OPR3	I always ensure strict compliance with the recommendations from previous output reviews.	N=140 (57.3%)	N=100 (40.7%)	N=2 (0.8%)	N=2 (0.8%)	N=1 (0.4%)	4.22	0.719
OPR4	In terms of feedback, I receive constructive remarks from my supervisor about my task output.	N=161 (65.6%)	N=65 (26.6%)	N=16 (6.6%)	N=2 (0.8%)	N=1 (0.4%)	4.08	0.737
OPR5	Regular opportunities to review my output are always given by my superiors.	N=89 (36.5%)	N=152 (62.2%)	N=1 (0.4%)	N=1 (0.4%)	N=1 (0.4%)	4.14	0.804
OPR6	The report on my output review is used by me to improve in areas that need improvement.	N=97 (39.4%)	N=127 (51.9%)	N=16 (6.6%)	N=4 (1.6%)	N=1 (0.4%)	4.18	0.778
OPR7	During meetings for output review, there is always an agreement between me and my supervisors on action plans affecting my output.	N=103 (41.9%)	N=115 (46.9%)	N=25 (10.4%)	N=1 (0.4%)	N=1 (0.4%)	4.44	0.598
OPR8	I feel much more comfortable with the output review process in my school.	N=140 (57.3%)	N=81 (33.2%)	N=2 (0.8%)	N=20 (8.3%)	N=1 (0.4%)	4.33	0.561
OPR9	I’m comfortable with the output review process in my school.	N=169 (169.9%)	N=38 (15.4%)	N=2 (0.8%)	N=3 (1.2%)	N=34 (13.7%)	4.33	0.561
	Average	N=120 (48.9%)	N=106 (43.1%)	N=11 (4.52%)	N=4 (1.6%)	N=5 (1.9%)	**4.225**	

Key: (OPR)= output planning, SA (5)=Strongly Agree, A (4)=Agree, N (3)=Neutral in Agreement, D (2)=Disagree, SD (1)=Strongly Disagree. (X) Descriptive means, St,dvt=Standard Deviation. Descriptive mean (2.00-2.99) low, Mean (3.00-3.99) high, and mean (4.00-5.00) very high.

Table 5 revealed regression analysis for the study, where the influence output review variable contributes 71.5% to Teachers’ Job Output in Secondary Schools in Kasese District. This is shown by the results in Table 4.10, where $R^{2}=0.715$ , F=16.648, and the standard error of the estimate Se=0.2524 . The descriptive analysis, which yielded a pooled mean of $\bar{x}=4.225,$ is as high as the legend for Table 4. Regression analysis revealed an R ^2^ =0.715 , suggesting a strong positive relationship between the influence of output review on teachers’ job output. Therefore, the null hypothesis is rejected based on the value of regression $R^{2}$ Fvalue given as F=16.648 in Table 6, since $F=16.548>F_{0.05,2,242}=3.00$ . This means that output reviewing has a significant influence on teachers’ job output in secondary schools in Kasese District, where $R^{2}=0.715$ . A large portion of the changes in teacher output can be attributed to the process of reviewing their performance. The contradiction of the hypothesis, which was the stated hypothesis, “Output reviewing has no significant influence on teachers’ job output in secondary schools in Kasese District,” is rejected.

**Table 5.** Summary of regression diagnostics.

Bayes factor	R	RSquare	AdjustedRSquare	Standard error of estimate	F
2.569E + 42	0.846	0.715	0.685	0.2524	16.648

**Table 6.** Bayes’ factor inference on pairwise correlations.

		Teachers job output	Output reviewing
Teachers Job Output	Pearson Correlation	1	0.321
	^a^Bayes Factor		0.00
	N	245	245
Output Reviewing	Pearson Correlation	0.321	1
	^a^Bayes Factor	0.00	
	N	245	245

^a^ Bayes factor: Inference on Pairwise Correlations.

A Pearson correlation coefficient of 0.321 indicates a weak-to-moderate positive association between teacher job output and output review, as shown in Table 6. Using Bayesian inference, the Bayes factor shows weak to moderate evidence in favour of the alternative hypothesis ( $H_{01}$ : a exists relationship), even though the correlation is low.

## Discussion

The study assessed the role of output monitoring and output review in influencing teachers’ job output in secondary schools in Kasese District. The findings indicate that both factors contribute significantly to teachers’ job performance, with output review exerting a greater impact than output monitoring. The findings on output monitoring revealed a very high level of agreement among teachers regarding the effectiveness of monitoring practices, with a pooled mean of 4.225 as depicted in Table 1. The majority of respondents strongly agreed or agreed that school management employs acceptable monitoring procedures (M = 4.48), that supervision techniques are comfortable (M = 3.93), and that monitoring has improved their teaching activities (M = 4.08). The objectivity of monitoring activities was highly rated (M = 4.44), and teachers expressed high satisfaction with the monitoring methods used by their supervisors **(**M = 4.33 **)**. The positive perception of monitoring procedures among teachers may be attributed to structured and transparent supervision practices that promote fairness, accountability, and professional growth. ^14^ The high ratings suggest that school management employs objective and supportive monitoring methods, creating a conducive work environment where teachers feel valued and motivated. Additionally, clear expectations, regular feedback, and constructive evaluations likely contribute to improved teaching practices and job satisfaction. ^15^ The result from this study supports other findings by, ^16, 17^ who emphasized that transparent and structured supervision fosters teacher engagement and improves instructional quality.

Statistically, the study revealed a moderate positive correlation (r = 0.521) between output monitoring and teachers’ job output as presented in Table 2. The regression analysis in Table 3 further confirmed that output monitoring contributes 58.9% (R ^2^ = 0.589) to teachers’ job output, with a statistically significant F-value (F = 14.071). These results indicate that schools with structured and objective monitoring frameworks have better teacher productivity, supporting previous studies by, ^3, 4^ which found that monitoring mechanisms enhance teachers’ accountability and instructional effectiveness. However, despite its positive impact, output monitoring alone does not fully explain variations in teacher performance, suggesting the need for additional interventions such as structured performance feedback and professional development opportunities. ^18^ Besides, the findings on output review indicate a stronger impact on teachers’ job output compared to output monitoring. The descriptive analysis revealed a very high level of agreement (pooled mean = 4.23), as depicted in Table 4. 92% of teachers agree or strongly agree that output reviews provide constructive feedback, improvement opportunities, and alignment with performance standards. The highest-rated statements included constructive feedback from supervisors (M = 4.48) and agreement on action plans during reviews (M = 4.44), demonstrating that teachers highly value structured performance evaluations as a tool for continuous improvement. These findings align with research by, ^19, 20^ which showed that teachers who receive regular, constructive performance reviews exhibit greater instructional effectiveness and student engagement. The stronger impact of output review on teacher job performance is likely due to its structured and constructive nature, which provides clear feedback, actionable improvement strategies, and alignment with performance standards. ^21^ Teachers highly value supervisory feedback and collaborative action plans as they promote professional growth, accountability, and instructional effectiveness. Regular performance reviews enhance motivation, refine teaching methods, and improve student engagement, making them more effective than passive monitoring. ^22^

Moreover, regression analysis presented in Table 5 revealed that output review accounts for 71.5% (R ^2^ = 0.715) of the variance in teachers’ job output, with a statistically significant F-value (F = 16.648). The Pearson correlation analysis presented in Table 6 showed a moderate positive relationship (r = 0.321) between output review and teachers’ job output, suggesting that while the correlation is not very strong, output review plays a substantial role in explaining teacher performance. ^23^ These results reinforce previous research by, ^19, 24^ which found that structured and data-driven performance evaluations significantly improve teaching quality and student outcomes among secondary school students. Furthermore, comparing the two factors, output review has a greater influence on teachers’ job output (R ^2^ = 0.715) compared to output monitoring (R ^2^ = 0.589). This suggests that while monitoring provides a framework for assessing teacher performance, structured performance reviews are more critical for driving improvements in teaching effectiveness. ^25, 26^ The statistically significant F-values (F = 16.648 for output review and F = 14.071 for output monitoring) indicate that both factors play a vital role in shaping teacher productivity, but output review has a more substantial impact. These findings suggest that school administrators should prioritize structured performance reviews over mere monitoring activities. ^27^ While monitoring helps track teacher performance, the actual process of reviewing and providing feedback is what drives improvements in instructional practices. Schools should consider the following strategies:

○ Implement regular performance review meetings where teachers and supervisors collaboratively discuss performance outcomes and improvement plans, ensuring that feedback is specific, constructive, and actionable, allowing teachers to use it for continuous improvement.
○ Link performance reviews with personalized professional development programs, ensuring that identified gaps are addressed through targeted training initiatives.
○ Incorporate self-assessments and peer evaluations into the review process to enhance engagement and accountability.

These recommendations align with the best practices in teacher evaluation outlined by, ^28– 30^ who emphasized that teachers who receive consistent, high-quality feedback demonstrate better instructional outcomes and student achievement. The null hypothesis (H _0_: Output monitoring and review have no significant influence on teachers’ job output in secondary schools in Kasese District) was tested. The findings indicate that output monitoring has a moderate effect on job output (R ^2^ = 0.589, F = 14.071, p < 0.05), leading to a partial rejection of the null hypothesis. However, the output review exhibited a stronger influence (R ^2^ = 0.715, F = 16.648, p < 0.05), resulting in a full rejection of the null hypothesis. These results suggest that while monitoring is essential for tracking performance, structured output reviews provide a more robust mechanism for improving teacher effectiveness.

## Conclusion

This study establishes that structured output monitoring and review play a significant role in enhancing teachers’ job performance in secondary schools in Kasese District. The findings confirm that while monitoring provides a framework for assessing performance, output review is more influential in driving improvements in teaching effectiveness. Given this, schools should integrate structured performance evaluations with clear feedback mechanisms to enhance teacher accountability and instructional quality. To improve teacher performance, education policymakers and school administrators should prioritize structured output reviews, linking them to professional development and targeted interventions. Additionally, adopting objective and transparent evaluation criteria can enhance teachers’ motivation and retention. Strengthening these mechanisms will ensure a sustainable and effective teacher evaluation system, ultimately improving student learning outcomes.

### Recommendations

Based on the findings of this study, the following recommendations are proposed to enhance teacher job performance in secondary schools in Kasese District:

○ Schools should establish consistent and transparent monitoring frameworks to track teacher performance objectively. Regular performance reviews should be institutionalized, ensuring teachers receive constructive and actionable feedback for continuous improvement.
○ Performance evaluations should be linked to personalized training programs, allowing teachers to address specific areas of improvement. Schools should organize periodic capacity-building workshops focused on lesson planning, instructional strategies, and classroom management.
○ Supervisors should provide specific, data-driven, and timely feedback to help teachers improve their instructional practices. Peer reviews and self-assessment tools should be incorporated to encourage teacher engagement and self-improvement.
○ Schools should implement clear and objective evaluation criteria to prevent bias and subjectivity in teacher assessments. Mechanisms should be introduced to minimize the administrative burden on teachers during monitoring and review processes.
○ Policymakers should introduce incentives for teacher retention, such as financial rewards, career progression opportunities, and improved working conditions. Schools in rural areas should receive additional support in the form of housing incentives and professional development opportunities to address teacher shortages.
○ Schools should adopt digital tools for efficient and objective teacher performance tracking, enabling data-driven evaluations.

By implementing these recommendations, schools and policymakers can strengthen output monitoring and review systems, ultimately improving teacher performance and student learning outcomes in secondary schools in Kasese District.

## Ethical considerations

The research team was given an introductory letter, and the collected data ensured the confidentiality of the information obtained. The data collected was used for the purpose for which it was collected. The researchers had to get the consent of the respondents and maintain their confidentiality. The names and personal identities of participants were not put on the instrument. The researchers received approval on 12/06/2024 with the approval no as KIU-2024-309, from the Research Ethical Review Committee of Kampala International University and the Uganda National Council for Science and Technology, UNCST.

## Informed consent to participate

All head teachers, deputy head teachers, directors of studies, and teachers in Kasese District who participated in this study provided their consent by signing a written informed consent form. We confirmed that all the participants signed the informed consent form. The study did not hurt the head teachers, deputy head teachers, directors of studies, teachers, or the schools.

## Data Availability

The authors declared the availability of the data used in the research. The data was deposited in the OSF database with the link: https://osf.io/skcj6/. Also included in the data availability is the DOI, https://doi.org/10.17605/OSF.IO/SKCJ6 (Asiati, Bagiwa, Muhammad, & Aja, 2025). ^31^ Data are available under the terms of the Creative Commons Attribution 4.0 International license (CC-BY 4.0).
