# Supplementary material for: The Role of Output Monitoring and Review on Teacher Job Performance in Secondary Schools: Evidence from Kasese District, Uganda
Source: F1000Res. 2025 Aug 4;14:764. [Version 1] doi: 10.12688/f1000research.166552.1 (PMC12413610; doi:10.12688/f1000research.166552.1)
Supplement: Peer review history

# Reviewer report: Reviewer response for version 1

**DOI:** 10.5256/f1000research.183555.r403086

**Published:** 2025-09-05

**Authors:** Muhammad Imran, Nazir Ahmed

**Affiliations:** 1 Shaheed Zulfiqar Ali Bhutto Institute of Science & Technology, Sindh, Pakistan; 2 Teacher Education, Federal Urdu University of Arts Sciences and Technology, Karachi, Sindh, Pakistan

Dear, editor overall quality of the article "The Role of Output Monitoring and Review on Teacher Job Performance in Secondary Schools: Evidence from Kasese District, Uganda" accepted for further process of indexing. Introduction, Literature, methodology and results dections are well explained. Need to improve Discussion part if 2nd reviewer also suggested improvement, otherwise accepted as it is.

Is the work clearly and accurately presented and does it cite the current literature?

Yes

If applicable, is the statistical analysis and its interpretation appropriate?

Yes

Are all the source data underlying the results available to ensure full reproducibility?

Yes

Is the study design appropriate and is the work technically sound?

Yes

Are the conclusions drawn adequately supported by the results?

Partly

Are sufficient details of methods and analysis provided to allow replication by others?

Yes

Reviewer Expertise:

Educational Leadership and Management

We confirm that we have read this submission and believe that we have an appropriate level of expertise to confirm that it is of an acceptable scientific standard.

# Reviewer report: Reviewer response for version 1

**DOI:** 10.5256/f1000research.183555.r403085

**Published:** 2025-09-05

**Authors:** Yari Dwikurnaningsih, Stefanus Christian Relmasira

**Affiliations:** 1 Faculty of Education, Satya Wacana Christian University, Salatiga, Central Java, Indonesia

1. Summary of the Article

 This article investigates the impact of output monitoring and output review on the job performance of secondary school teachers in Kasese District, Uganda. The study addresses a critical issue within the Sub-Saharan African educational context, directly linking teacher performance management to the quality of student learning outcomes. Using a quantitative approach with a sample of 245 respondents, the study employs Pearson correlation, linear regression, and Bayesian inference to analyze the data. The findings reveal that both output monitoring and output review significantly and positively influence teacher job performance, with output review having a comparatively stronger impact. The paper concludes by offering several concrete recommendations for policymakers and school administrators, such as digitizing teacher evaluations and linking performance reviews to professional development.

 2. Strengths of the Article

 The manuscript has several notable strengths that contribute to the field of educational management:

- High Relevance and Timeliness: The research addresses a topic of significant importance. The effectiveness of teacher performance management is a crucial determinant of educational quality, making this study highly relevant for educators, administrators, and policymakers, particularly in developing countries.
- Methodological Analysis: The use of statistical approach, including linear regression, Pearson correlation, and Bayesian inference to test for robustness, strengthens the validity and reliability of the findings.
- Validated Instrumentation: The credibility of the collected data is well-supported by the use of validated research instruments. The authors report a Content Validity Index (CVI) of ≥0.7 and a Cronbach’s Alpha reliability test (≥0.7) following a pilot study, ensuring the quality of the measurement tool.
- Actionable and Practical Recommendations: The article proposes clear, concrete, and applicable recommendations. Suggestions such as digitizing teacher evaluations, providing review-based training, and offering incentives are directly relevant to policy and can be implemented to improve educational systems.

3. Areas for Improvement

 While the study is fundamentally sound, several areas could be improved to enhance its clarity, scientific rigor, and impact. The following constructive suggestions are based on the provided draft review:

 1. Inconsistency in Research Design Description

- Issue: There is a significant discrepancy between the abstract and the methodology section. The abstract states that the study "integrating quantitative and qualitative approaches" and used "interview guides." However, the methodology section describes the research approach as solely "quantitative" and only details the use of a structured questionnaire. No qualitative data from interviews is presented or discussed in the results.
- Suggestion: The authors must clarify this inconsistency. If a mixed-methods design was indeed used, the qualitative findings should be elaborated upon narratively (e.g., through specific case studies or thematic analysis of interview data) to provide a richer context. If the study was purely quantitative, all references to a qualitative approach and interview guides must be removed from the abstract to accurately reflect the study's design.

2. Lack of Detail in Instrumentation

- Issue: The manuscript does not provide sufficient detail about the questionnaire itself. The specific aspects, indicators, and sample items used to measure the variables (output monitoring, output review, and teacher job performance) are not described.
- Suggestion: To improve transparency and allow for replication by other researchers, the authors should include an appendix or a table that outlines the instrument's structure. This would give readers a clearer understanding of how the core concepts were operationalized.

3. Limited Sample Description and Generalizability

- Issue: The paper does not provide a detailed breakdown of the final sample. While the total number of respondents (245) is mentioned, the specific number of head teachers, deputy head teachers, directors of studies, and teachers who participated is not specified.
- Suggestion: The authors should include a table detailing the composition of the sample. Additionally, the authors should more explicitly acknowledge the limitations of generalizability in the discussion section, given that the study was conducted in a single district. This would provide a more nuanced understanding of the scope of the findings.

4. Conclusion

 Overall, this is a valuable and well-executed study that contributes important insights into teacher performance management in Uganda. The strengths lie in its relevant topic, analysis, and practical recommendations. By addressing the points raised above—particularly the inconsistency in the stated research design and the lack of detail regarding the instrument and sample—the authors can significantly enhance the scientific quality and clarity of their manuscript.

Is the work clearly and accurately presented and does it cite the current literature?

Partly

If applicable, is the statistical analysis and its interpretation appropriate?

Yes

Are all the source data underlying the results available to ensure full reproducibility?

Partly

Is the study design appropriate and is the work technically sound?

Partly

Are the conclusions drawn adequately supported by the results?

Yes

Are sufficient details of methods and analysis provided to allow replication by others?

Partly

Reviewer Expertise:

educational supervision, leadership, training in education, evaluation of educational program and policy

We confirm that we have read this submission and believe that we have an appropriate level of expertise to confirm that it is of an acceptable scientific standard, however we have significant reservations, as outlined above.

# Reviewer report: Reviewer response for version 1

**DOI:** 10.5256/f1000research.183555.r403084

**Published:** 2025-08-28

**Authors:** Sehan Rifky

**Affiliations:** 1 Syekh-Yusuf Islamic University (Ringgold ID: 175493), Tangerang, Banten, Indonesia; 2 State Cyber Islamic University Syekh Nurjati Cirebon, Cirebon, West Java, Indonesia

The article titled *"The Role of Output Monitoring and Review on Teacher Job Performance in Secondary Schools: Evidence from Kasese District, Uganda"* is clearly written and methodologically sound. The study employs a cross-sectional design, integrating appropriate quantitative analyses, including Pearson correlation, regression, and Bayesian inference.

 The article presents a well-structured narrative, supported by recent and relevant literature. The methodology is detailed, including sample design, instrument validation, and ethical considerations. All source data are openly available, ensuring transparency and replicability.

 Summary:

 The study explores the influence of output monitoring and review on teacher performance in Kasese District, Uganda. Findings reveal that while both factors significantly impact performance, output review (R² = 0.715) exerts a greater influence than monitoring (R² = 0.589). Teachers who receive structured feedback show higher motivation, instructional quality, and engagement.

 Constructive Feedback:

 While most elements are solid, a few areas require clarification:

1. In the hypothesis section, “output planning” is mentioned but not analyzed in the results. Clarify its role or remove it for consistency.
2. Terms like “performance appraisal” and “output review” are used interchangeably; authors should define whether these are conceptually distinct or the same.
3. The explanation of Bayesian inference would benefit from a brief interpretation of the Bayes factor values reported.

These minor revisions would further strengthen the article's scientific rigor and clarity.

Is the work clearly and accurately presented and does it cite the current literature?

Yes

If applicable, is the statistical analysis and its interpretation appropriate?

Yes

Are all the source data underlying the results available to ensure full reproducibility?

Yes

Is the study design appropriate and is the work technically sound?

Yes

Are the conclusions drawn adequately supported by the results?

Yes

Are sufficient details of methods and analysis provided to allow replication by others?

Yes

Reviewer Expertise:

Educational Leadership and Policy | Teacher Performance Evaluation | Public Sector Management | Educational Administration | Research Assessment Methodologies

I confirm that I have read this submission and believe that I have an appropriate level of expertise to confirm that it is of an acceptable scientific standard, however I have significant reservations, as outlined above.
